# Supplementary material for: Maternal Magnolol Supplementation during Pregnancy and Lactation Promotes Antioxidant Capacity, Improves Gut Health, and Alters Gut Microbiota and Metabolites of Weanling Piglets
Source: Metabolites. 2023 Jun 27;13(7):797. doi: 10.3390/metabo13070797 (PMC10383630; doi:10.3390/metabo13070797)
Supplement: Supplementary file 1 [file metabolites-13-00797-s001.zip › Table S1. Nutrients content of the control diet and pre-starter.pdf]

Table S1. Nutrients content of the control diet and pre-starter (%).

Nutrient Levels	Content		
	**Pregnancy**	**Lactation**	**Pre-starter**
Dry matter	88.00	88.00	92.00
Crude protein	14.00	16.00	23.00
Crude fiber	8.00	6.00	6.00
Crude Ash	10.00	9.00	6.50
Total phosphorus	0.43	0.43	0.73
Calcium	0.95	0.95	0.78
NaCl	0.55	0.55	0.43
Lysine	0.55	0.85	1.70
